# What are the odds of anxiety disorders running in families? A family study of anxiety disorders in mothers, fathers, and siblings of children with anxiety disorders

**DOI:** 10.1007/s00787-017-1076-x

**Published:** 2017-11-06

**Authors:** Liesbeth G. E. Telman, Francisca J. A. van Steensel, Marija Maric, Susan M. Bögels

**Affiliations:** 1 Research Institute of Child Development and Education, University of Amsterdam, Postbus 15780, 1001 NG Amsterdam, The Netherlands; 2 Department of Developmental Psychology, University of Amsterdam, Amsterdam, The Netherlands; 3 UvA minds, Academic Outpatient Child and Adolescent Treatment Center, Amsterdam, The Netherlands

**Keywords:** Anxiety disorders, Children, Parents, Siblings, Family

## Abstract

This family study investigated (1) the prevalence of anxiety disorders (ADs) in parents and siblings of children (*n* = 144) aged 8–18 years with ADs compared to control children (*n* = 49), and (2) the specificity of relationships between child–mother, child–father, and child–sibling ADs. Clinical interviews were used to assess current DSM-IV-TR ADs in children and siblings, and lifetime and current ADs in parents. Results showed that children with ADs were two to three times more likely to have at least one parent with current and lifetime ADs than the control children (odds ratio (OR) = 2.04 and 3.14). Children with ADs were more likely to have mothers with current ADs (OR = 2.51), fathers with lifetime ADs (OR = 2.84), but not siblings with ADs (OR = 0.75). Specific relationships between mother–child ADs were found for Social Anxiety Disorder (SAD, OR = 3.69) and Generalized Anxiety Disorder (OR = 3.47). Interestingly, all fathers and siblings with SAD came from families of children with SAD. Fathers of children with SAD were more likely to have lifetime ADs themselves (OR = 2.86). Findings indicate that children with ADs more often have parents with ADs, and specifically SAD is more prevalent in families of children with SAD. Influence of parent’s (social) ADs should be considered when treating children with ADs.

## Introduction

One of the well-examined features of anxiety disorders (ADs) is that they tend to ‘run in families’ and are transmitted from parents to children [1]. So far, evidence is available that this can partly (30–40%) be attributed to biological/genetic markers [2]. However, a recent study in a large sample of children of twins found evidence for direct environmental transmission of ADs from the parent to the child, which could imply that parental anxiety shapes the way for a parenting style that contributes to the development of ADs in children [3]. One of these parenting styles is over-controlling behavior, which limits children’s autonomy, and could maintain their inhibition and anxiety. Moreover, children’s anxious behavior could also evoke parents' over-controlling parenting behavior in order to prevent distress in their child [4]. In addition, parents’ anxious behavior and expressed anxiety could also promote and maintain child anxiety through modeling, i.e. catastrophizing and over-attribution of threat [1, 4, 5]. Together this shows that the development and maintenance of ADs in children are a result of genetic, environmental and parenting factors.

In order to inform our understanding of the etiology, prevention and treatment of child ADs, it is important to know to what extent parents of children with ADs have ADs themselves. It has been shown that children with ADs did less well in treatment when their mothers had ADs [6], and CBT for children with AD is less effective when parents have an AD themselves [7]. Numerous studies have examined the familial risk of ADs; however, most used a top-down approach (i.e., examining the presence of ADs in the offspring of parents with ADs) [2, 8, 9]. Another way to examine transmission of ADs is to use a bottom-up approach: evaluating the presence of ADs in parents of children that are diagnosed with ADs.

Bottom-up studies have shown increased rates of ADs in parents of children referred with ADs compared to parents of typically developing children, with lifetime prevalence rates ranging between 46 and 83% in mothers, and between 30 and 45% in fathers [10–13], compared to 15–38% in parents of typically developing children. While these initial studies were valuable in terms of examining the associations between child and parental ADs, it should also be noted that some of these studies included participants diagnosed with DSM-III disorders [13, 14], had low father participation and/or did not use a diagnostic instrument to assess fathers’ clinical status [10, 11, 13], or lacked a control group [11]. In addition, an important aspect that was not addressed in these studies is the prevalence of ADs in siblings.

To determine familial transmission of ADs and to understand the mechanisms behind it, it is important to include the whole family. Recently, fathers are more often included in child anxiety research, and it has been suggested that fathers’ ADs contribute differently to the development of child anxiety than mothers, for example father’s role in physical play may be affected more by his own anxiety (disorders) than mother’s role of caring [15, 16]. Nevertheless, associations between mother–child ADs seem to be stronger than father-child ADs, with odds ratios of lifetime ADs found between 3.40 and 4.44 for mothers and odds ratios between 2.33 and 3.17 for fathers [10, 12]. However, Last et al. [14] found twice as many ADs in fathers and brothers (male relatives combined) of children with ADs than in control children, and no difference in mothers’ prevalence, but note that father participation was low in the group of children with ADs (38%). To conclude, the relative risks of mothers versus fathers of children referred with ADs being affected by own ADs need to be established in studies in which no more fathers are missing than mothers, in order to draw valid conclusions, as missing fathers are likely to be systematically missing (e.g., because of divorce, anxiety disorders, or busy in the outside world [16]).

Next to the increased prevalence of ADs in mothers and fathers, siblings of children with mental health problems are at risk of developing psychopathology themselves [17]. However, it is unclear whether they develop an AD more often than siblings of typically developing children. Only a few studies examined the prevalence of ADs in siblings of anxious children [14, 18–20]. The study of Dia and Harrington [18] showed that 12% of siblings of children with ADs had a (mostly mother-reported) diagnosis of AD, but this study did not include a control group and two other studies examining anxiety in siblings lacked a DSM-based diagnostic assessment [19, 20]. One study did use a control group and a diagnostic instrument (but based on DSM-III), and found that siblings of children with ADs were about twice as likely to have ADs as siblings of typically developing children [14].

In addition to the intergenerational transmission of ADs, it is interesting to examine to what extent there is diagnostic specificity in this transmission. Diagnostic specificity suggests that children of anxious parents are at greater risk of developing the same AD as their parents, because their parents, next to heritability aspects [2], model or communicate their specific anxieties to their children [12]. A better understanding of the specificity of the transmission of ADs could inform child treatment or preventive interventions. In previous studies, some evidence has been found for specificity of social anxiety disorder (SAD), separation anxiety disorder and panic disorder between child and mother, but not between child and father [10, 12]. Otherwise, strong associations have been found between parental social phobia and social phobia in their adolescent children (odds ratio = 4.7; [21]). In addition, specificity in siblings was found for agoraphobia and social phobia [20], although those ADs were not measured with a diagnostic instrument but based on clinical judgment. In contrast, other studies did not find evidence for specificity, thus providing support for a transmission of anxiety in general and not for specific ADs, which implies that ADs have shared etiology [3, 10]. Thus, only few studies have studied specificity of ADs between family members and those studies that did, found contrasting results.

Finally, considering the comorbidity between ADs and depressive disorders [22], parents of children with ADs may be more likely to have depressive disorders than parents of children without ADs. Bottom-up studies have found mixed results, as Cooper et al. [10] found that children with ADs were more likely to have mothers with current and lifetime depressive disorder than children without anxiety disorders, whereas Last et al. [14] and Hughes et al. [12] did not find that parents of children with ADs were at increased risk for lifetime mood disorders. In addition, stronger associations have been found between ADs in children and parents than between ADs in children and depressive disorders in parents [8]. Moreover, it remains unclear whether children with ADs have an elevated risk of having parents with depressive disorder, or that this is mainly due to an elevated risk of ADs in parents with comorbid depressive disorder [23]. Thus, it is important to consider comorbid depressive disorder in parents when examining ADs in parents of children with ADs.

The main aim of the current study was to examine the transmission of ADs in the family using a bottom-up approach, in order to understand to what extent children with ADs grow up with parents and siblings with ADs. Establishing the risk for ADs in mothers, fathers, and siblings of referred children with ADs is important for our understanding of the etiology, prevention, and treatment of child ADs. In this study, we examined the association between ADs in a clinically referred sample of children with ADs, their mothers, fathers, and siblings, using diagnostic interviews, and we compared these rates to a control group of typically developing children. We hypothesized that mothers, fathers, and siblings of children with ADs would be more likely to have ADs than families of typically developing children. Additionally, given that parental depression has been associated with the development of child ADs, we examined the association between depressive disorder in parents of children with AD and parents of children in the control group. Finally, we examined specificity of transmission, i.e. whether a specific AD in the child (e.g., SAD) would be associated with the same AD (SAD) in the parent or not; and familial risk of specific ADs, i.e. whether children with a specific AD (e.g., SAD) would be more likely to have parent(s) and/or siblings with any AD, compared to children with other ADs.

## Methods

### Participants and procedure

In total, 193 children participated: 144 children with ADs and 49 typically developing children (further referred to as controls). Reports were present from 191 (99.0%) mothers and 172 (89.1%) fathers. In total, 162 families (83.9%) had more than one child (total number of siblings = 239). Out of the 239 siblings, 168 siblings from 123 (75.9%) families participated; of those who did not participate, 33 siblings (13.8%) did not fulfill the age range of 7–23 years, 4 siblings (1.7%) did not live at home anymore, and 44 siblings (14.2%) did not want to participate or were unable to reach. Table 1 shows the characteristics of both groups.

**Table 1:** Demographics of participants

	Children with ADs (*n* = 144)	Controls (*n* = 49)
Age (*M*, SD)	12.38 (2.71)	12.44 (2.63)
Number (%) of boys	57 (40.6%)	21 (44.7%)
Married families (*n*, %)	113 (78.5%)	35 (71.4%)
Parental age (*M*, SD)		
Mother	41.95 (4.92)	42.58 (5.51)
Father	44.97 (5.04)	44.77 (5.40)
Parental educational level^a^ (*M*, SD)		
Mothers**	5.06 (1.96)	6.17 (1.81)
Fathers*	5.69 (2.03)	6.44 (2.02)
Siblings	*n* = 130	*n* = 38
Age**	13.90 (4.16)	11.86 (2.83)
Number (%) of boys*	62 (48.1%)	25 (67.6%)

* *p* < 0.05, ** *p* < 0.01

^a^ On a scale of 1 (no education) to 9 (university)

Participants were drawn from a sample of children recruited for a study on the relation between anxiety and parental rearing [24], and most were enrolled in a study on the efficacy of CBT (see [7] for inclusion criteria). A community sample of control children was recruited through advertisements in journals and magazines and received a €50 fee [7]. Children were included if they did not receive any support from a child mental health care center. Note however that eight controls (16.3%) met the criteria for an AD on the Anxiety Disorders Interview Schedule-Child and Parent Versions (ADIS-C/P) [25] (see Table 2). It was decided to keep these children in the control sample, as this percentage does not deviate from prevalence rates found in other studies of typically developing children [26]. Moreover, in practice only 30% of children that have an AD are referred to mental health care centers and referral is associated with the impairment of the child [27, 28]. Of note, analyses were performed with and without these children and are reported in the results section.

**Table 2:** Prevalence of lifetime anxiety disorders of families of children referred with ADs and typically developing children

	Children referred with ADs (*n* = 144)								Typically developing children (*n* = 49)							
	Child		Mother (*n* = 134)		Father (*n* = 124)		Siblings (*n* = 130)		Child		Mother (*n* = 49)		Father (*n* = 38)		Siblings (*n* = 38)	
	*N*	%	*N*	%	*N*	%	*n*	%	*n*	%	*n*	%	*N*	%	*n*	%
Any AD	144	100	73	54.5	36	29.0	34	26.2	8	16.3	20	40.8	4	10.5	12	31.6
SAD	99	68.8	37	27.6	15	12.1	15	11.5	2	0	8	16.3	4	10.5	6	15.8
GAD	74	51.4	28	20.9	18	14.5	13	10.0	1	4.1	8	16.3	1	2.6	3	7.9
SP	83	57.6	48	35.8	14	11.3	17	13.1	6	12.2	4	8.2	0	0	8	21.1
PAG	32	22.2	30	22.4	14	11.3	2	1.5	0	0	4	8.2	0	0	0	0
SEP	62	43.1					4	3.1	0	0					0	0
OCD	7	4.9	3	2.2	2	1.6	3	2.3	0	0	1	2.0	0	0	0	0
PTSD	8	5.6	15	11.2	3	2.4	2	1.5	0	0	2	4.1	0	0	0	0
Average number of ADs	M 2.53	SD 1.28	M 0.95	SD 1.06	M 0.5	SD 0.92	M 0.44	SD 0.75	M 0.18	SD 0.44	M 0.49	SD 0.68	M 0.03	SD 0.16	M 0.48	SD 0.64

*AD* anxiety disorder, *SAD* social anxiety disorder, *GAD* generalized anxiety disorder, *SP* specific phobia, *PAG* panic disorder and/or agoraphobia, *SEP* separation anxiety disorder, *OCD* obsessive-compulsive disorder, *PTSD* posttraumatic stress disorder

Clinical interviews were carried out by trained psychology research assistants, who had received 2 days training from the authors of the Dutch translation of the ADIS-C/P [29]. Their diagnostic ratings had to match those of experienced interviewers from Temple University. Moreover, each research assistant videotaped four of her own interviews, which were rerated by two trained students. The total interrater agreement for all ADIS diagnoses (κ) was based on the presence or absence of the specific anxiety disorder; ADIS-Child report 0.89, ADIS-Parent about child report 0.83, and ADIS-Adult report 0.94. The interviewer was blind to the goals of this study. Medical-ethical approval was obtained and all participants signed informed consent.

## Measures

### Anxiety disorders children and siblings

Anxiety disorders for children and siblings were assessed with the ADIS-C/P [25], which follows criteria of the DSM–IV [30]. The interview was conducted separately with the child and with both parents together when the parents reported about their child(ren). Father and mother reported separately about their own ADs. The interview starts by examining symptoms of ADs, and when this symptom criterion is fulfilled, the respondent is asked to rate the impairment for daily functioning on a 0–8 point scale. When a score of 4 or higher is given, then an AD is assigned. Child and parent interview scores were aggregated based on recommendations of the manual in which the aggregated rating consisted of a composite score of the combined parent and child interview [25]. Psychometric properties of the ADIS-C/P are good [31]. Interrater reliability for this sample was high, total interrater agreement (kappa) was 0.89 for child report, and 0.83 for combined parent report [24].

### Anxiety disorders parents

Parents’ ADs were assessed with the ADIS-IV-L [32] which is a structured interview that assesses DSM-IV disorders. Both current and lifetime diagnoses were gathered from the interview. In the interview, symptoms of disorders are checked, and when all criteria were met an impairment rating (on a scale 0–8) was given. A rating of 4 or higher indicates a diagnosis. Psychometric properties of the ADIS-IV-L are good [33] and interrater reliability for this sample was high (0.94, [24]).

### Statistical analyses

First, odds ratios for the clinically anxious children against controls were calculated for the risk of (1) having a parent (father and/or mother) with AD(s), (2) having a mother with AD(s) (3) having a father with AD(s), and (4) having a sibling with AD(s). Siblings were assigned to have an AD when one or more of the siblings had at least one AD (due to the small number of children with 2 or more siblings, data for siblings from the same family were collapsed). Second, odds ratios for the clinically anxious children against controls were calculated separately for having a mother or father with lifetime or current depressive disorder. Logistic regression was used to estimate the odds ratios, with group (children with ADs versus controls) as predictor and mother, father, or sibling AD as the dichotomous dependent variable. Parental education level was used as a covariate in the analyses because parents of children with ADs were found to have a lower educational level than parents of controls, and sibling age in the comparison of siblings of both groups. Wald test was used to evaluate predictors, and, in line with our hypotheses, *p* values were divided by two because of one-sided testing [34]. Odds ratios were calculated to examine the strength of the effect; the closer the odds ratio is to 0, the smaller the effect [35].

Second, following the study of Hughes et al. [12], the hypothesis of specific AD in families was tested within the group of children with ADs. Two approaches were used. In favor of the specificity hypothesis [12], odds ratios for each specific AD was calculated using logistic regression with the parent/sibling AD as the dependent variable, and the specific child AD as the predictor (e.g. child Generalized Anxiety Disorder (GAD) versus no GAD predicting parent GAD). In favor of a more familial risk of ADs, odds ratios for the risk of parents/siblings having ADs was calculated using logistic regression with the presence of ADs as the dependent variable, and the specific child AD (e.g., SAD, GAD) as the predictor. Wald test was used to evaluate predictors using two-sided testing, as specificity of ADs was an explorative question.

For the specificity analyses, maternal, paternal and sibling anxiety was assessed within the group of anxious children for five groups with the most prevalent child AD: social anxiety disorder (SAD), generalized anxiety disorder (GAD), specific phobia (SP), separation anxiety disorder (SEP), and panic disorder/agoraphobia (PAG). Due to high comorbidity among ADs [22, 26], which was also seen in the present study (see Table 2), odds ratios were calculated regardless of comorbidity. Thus, for example, the risk of SAD in mothers of children with SAD was calculated, regardless whether the child or mother had other ADs. In a few cases, one of the cells contained zero and therefore odds ratios could not be calculated. In those cases, a description of the prevalence rate instead of an odds ratio is given.

## Results

### Odds ratios for ADs running in the family

The prevalence of ADs for mothers, fathers and siblings is shown in Table 2. Having ADs as a child raised the odds of having at least one parent with (1) lifetime AD(s) (OR 2.04, 95% CI 1.02–4.06, *p* = 0.022), and (2) current ADs (OR 3.14, 95% CI 1.34–7.35, *p* = 0.005). Inspecting fathers and mothers separately, children with AD were not more likely to have mothers with lifetime AD(s) than controls (OR 1.29, 95% CI 0.63–2.63, *p* = 0.244), but more likely to have mothers that met criteria for current AD(s) (OR 2.51, 95% CI 1.01–6.21, *p* = 0.024). Children with ADs were significantly more likely to have fathers with lifetime AD(s) (OR 2.84, 95% CI 0.92–8.75, *p* = 0.035) and borderline more likely to have fathers with current AD(s) (OR 4.12, 95% CI 0.51–33.31, *p* = 0.092) compared to controls. Finally, children with ADs were not more likely to have siblings with AD(s) than controls (OR 0.75, 95% CI 0.29–1.93, *p* = 0.273).

Additionally, the prevalence of depressive disorder in the parents was examined. Lifetime depressive disorder was present in 29.9% of the mothers and 13.7% of the fathers in the clinical group; in the control group 26.5% of the mothers and 13.2% of the fathers had lifetime depressive disorder. Results showed that children with ADs were not more likely to have mothers with lifetime depressive disorder (OR 1.00, 95% CI 0.45–2.21, *p* = 0.499), and also not more likely to have fathers with lifetime depressive disorder (OR 1.15, 95% CI 0.38–3.44, *p* = 0.403) than controls. For current depressive disorder, a trend towards significance was found for mothers of children with AD compared to children of the control group (OR 5.35, 95% CI 0.67–42.65, *p* = 0.057), and no difference between children with ADs and controls was found for fathers (OR 0.26, 95% CI 0.03–1.99, *p* = 0.097).^1^

### Specificity and familial risk

Table 3 displays the results regarding parents’ and siblings’ risk for a specific (same) AD as predicted by the child’s specific AD. It was found that family members of children with SAD were more likely to have SAD themselves, that is (1) children with SAD were more likely to have mothers with lifetime SAD than children with other ADs (OR 3.69, 95% CI 1.20–11.40, *p* = 0.023), (2) odds ratios could not be calculated for father–child SAD because all fathers with lifetime SAD (17.4%, *n* = 15) had children with SAD, and (3) no odds ratios could be calculated for sibling–child SAD because all siblings with SAD (22.4%, *n* = 15) were from families with children with SAD. Finally, there was evidence of specificity for GAD: children with GAD were more likely to have mothers with lifetime GAD compared to children with other ADs (OR 3.47, 95% CI 1.28–9.41, *p* = 0.014). No specific risk was found for specific phobia, panic disorder and/or agoraphobia, and separation anxiety disorder.

**Table 3:** Results of specificity analyses: odds ratio and 95% confidence interval of child AD compared to lifetime mother, father, and current sibling AD

Child	Specificity					
	Mother AD^1^		Father AD^1^		Sibling AD^1^	
	OR	95% CI	OR	95% CI	OR	95% CI
SAD	3.69*	1.20–11.40	∞^a^		∞^a^	
GAD	3.47*	1.28–9.41	0.93	0.33–2.65	3.42	0.88–13.31
SP	1.11	0.50–2.49	0.61	0.19–1.92	3.11	0.91–10.59
PAG	2.43	0.91–6.45	1.28	0.25–6.60	∞^a^	
SEP					1.49	0.20–11.03

No specificity could be calculated for parents’ SEP because SEP was not measured with the ADIS-IV-L for parents

*AD* anxiety disorder, *SAD* social anxiety disorder, *GAD* generalized anxiety disorder, *SP* specific phobia, *PAG* panic disorder/agoraphobia, *SEP* separation anxiety disorder, *OR* odds ratio, *95% CI* 95% confidence interval

* p < 0.05

^1^ Each column represents the odds ratio of having the same AD as the child

^a^ ∞ = not applicable: odds ratio could not be calculated because one of the cells contained zero: all fathers/siblings with SAD came from families with children with SAD; all siblings with PAG came from families without children with PAG

Table 4 displays the results for parents’ and siblings’ familial risk of having any AD as predicted by the child’s specific AD. It was found that children with GAD were more likely to have mothers with lifetime ADs than children with other ADs (OR 2.48, 95% CI 1.23–4.98, *p* = 0.011). Children with SAD were more likely to have fathers with lifetime ADs (OR 2.86, 95% CI 1.07–7.60, *p* = 0.035), but children with specific phobia were less likely to have fathers with lifetime ADs (OR 0.41, 95% CI 0.19–0.90, *p* = 0.027).

**Table 4:** Odds ratio and 95% confidence interval of specific child AD compared to lifetime mother, father, and current sibling AD

Child AD	Any anxiety disorder					
	Mother any AD		Father any AD		Sibling any AD	
	OR	95% CI	OR	95% CI	OR	95% CI
SAD	1.60	0.77–3.33	2.86*	1.07–7.60	1.28	0.49–3.35
GAD	2.48*	1.23–4.98	0.57	0.26–1.25	0.83	0.35–1.98
SP	1.65	0.83–3.29	0.41*	0.19–0.90	1.69	0.71–4.07
PAG	2.03	0.84–4.90	1.76	0.68–4.54	0.53	0.18–1.61
SEP	0.57	0.29–1.14	0.99	0.45–2.16	1.43	0.60–3.44

*AD* anxiety disorder, *SAD* social anxiety disorder, *GAD* generalized anxiety disorder, *SP* specific phobia, *PAG* panic disorder/agoraphobia, *SEP* separation anxiety disorder, *OR* odds ratio, *95% CI* 95% confidence interval

* p < 0.05

## Discussion

The aims of the current study were to examine whether (I) children with ADs referred to community mental health care centers were more likely to have mothers, fathers, and siblings with ADs than typically developing children, and (II) children with specific ADs were more likely to have parents and siblings with the same AD (specificity) and/or parents and siblings with any AD (familial risk). Regarding (I), as compared to controls, children with ADs were respectively two and three times more likely to have at least one parent with lifetime and current ADs; two-and-a-half times more likely to have a mother with current ADs; nearly three times more likely to have a father with lifetime ADs, and—although borderline significant—four times more likely to have fathers with current ADs. Children with ADs were not more likely to have siblings with ADs. Regarding (II), specificity was found for mother–child SAD, all fathers with lifetime SAD had children with SAD, and all siblings with SAD were siblings from children who had SAD. In addition, specificity was found for mother–child GAD. At the same time, there were indications of a familial risk for some child ADs: (1) children with SAD were nearly three times more likely to have fathers with lifetime AD, and (2) children with GAD were two-and-a-half times more likely to have mothers with lifetime AD, compared to children with other ADs. Each of these findings is discussed in more detail below.

In line with our hypothesis and previous research [10, 12], we found that children with ADs were more likely to have mothers and fathers with ADs than controls. Results of the current study showed comparable sizes of odds ratios for mothers and fathers, whereas previous research mainly found stronger associations for internalizing problems between mother–child than for father–child [10, 36]. Therefore, our findings could suggest that both mothers and fathers are important to consider when assessing transmission of ADs in families. Moreover, we have found no heightened prevalence of depressive disorder in mothers and fathers of children with ADs compared to controls, which adds to the evidence that ADs are running in families. In contrast to our hypothesis, we did not find that siblings of children with ADs were more likely to have ADs which could be explained by the child susceptibility hypothesis. That is, some children, who are genetically susceptible for the development of an AD, because of an anxious temperament, are more likely to be affected by the consequences of living in a family with parental ADs than siblings who are not genetically-burdened, based on gene-environment interaction [37, 38]. In addition, anxious parents may be differentially susceptible for the anxious temperament of their child (for example experiencing the child as highly vulnerable), and consequently treat their anxious child different from their non-anxious children, for example by overprotecting their anxious child, which could influence parents’ risk of developing pervasive ADs [3]. Another, methodological, explanation for the lack of heightened risk of ADs in siblings of children with ADs, is the elevated prevalence of ADs in siblings of both groups (26.2% in the AD-group and 31.6% in the control group) when compared to the general prevalence of childhood AD (8.3–27%, [26]), as well as when compared to previous studies on siblings of children with AD (21.7%, [14]; 12%, [18]). It is remarkable that these rates are this high, and they are higher than would be expected in this sample. One explanation could be that the siblings that were interested in participating in our study were more familiar with anxiety symptoms. Moreover, the siblings of the control group were younger and consisted of more girls compared to the group of children with ADs, which could have affected prevalence rates [26]. Larger child and sibling samples are needed, as well as full participation of siblings to prevent systematic missing data, in order to examine the prevalence of ADs in siblings.

Strong evidence was found for the specificity of mother–child SAD, and for an elevated risk of SAD in fathers and siblings of children with SAD. These findings are interesting as they suggest that something specific is happening with the (familial) transmission of SAD. Next to heritability of an inhibited temperament (although only moderately, [39]), it could be that mothers and fathers of children with SAD are lacking certain social skills, which are modeled to the children and thus leading to childhood SAD. Previous studies have found that mother–child SAD is related [12], but the finding that all fathers of children with SAD have SAD themselves is interesting. This could be explained by the finding that socially anxious fathers show less challenging parenting behavior, which is a risk factor for the child developing social anxiety [40]. This indicates that fathers do have a different role in the development of ADs in their offspring than mothers (as suggested by Bögels and Phares [15]), and that father’s role may be particularly important for children with SAD [16, 41]. Interestingly, there are indications that children with SAD respond less well to treatment [42]. This could be related to the finding that SAD runs in the family and perhaps SAD needs a more family oriented treatment approach than other ADs. In sum, it seems that SAD runs in the family, and as SAD is the most common AD [43], more research towards the mechanisms of family transmission of SAD in specific is necessary.

Next to SAD, specificity was found for mother–child GAD, that is, children with GAD were more likely to have mothers with lifetime GAD than children with other ADs. This is interesting as previous research suggested that avoidance in GAD is less observable and therefore less easily transmitted through modeling [12]. Thus, it seems that GAD is transmitted through another mechanism, and it has been proposed that mothers transmit the cognitive styles associated with GAD, for example that worry helps to cope with uncertainty [44]. Next to this environmental transmission of GAD, it could be that genetic transmission of a certain trait or physiological marker is more important in GAD than in other ADs [2]. Furthermore, the specificity of both mother–child SAD and GAD could indicate a shared etiology or trans-diagnostic perspective, for example the tendency to worry that is present in both disorders [45]. Nevertheless, we cannot rule out that there are specific or common pathways in the transmission of SAD and GAD between mothers and children and vice versa. These effects should be examined bi-directionally in order to understand which mechanisms account for the specificity and to understand to what extent the worrying child affects maternal worry and maternal feelings of uncertainty.

Finally, we have found indications of a more familial risk of certain ADs, because children with GAD were more likely to have mothers with ADs, whereas children with SAD were more likely to have fathers with ADs, compared to children with other ADs. It could be that the mechanisms of transmission from father to child are different from transmission from mother to child, and/or that mothers and fathers respond differently to an anxious subtype in the child, i.e. a child with GAD evokes more anxiety in the mother while a child with SAD evokes more anxiety in the father. Previous studies have shown that fathers of socially anxious children show less emotional warmth and more rejection [41] and this relation is likely to be bi-directional, i.e. fathers show less warmth because their child is socially anxious and/or their child becomes more socially insecure because fathers show less warmth and more rejection [15]. Concerning GAD, it could be that mothers of children with GAD are transmitting their anxious thoughts and behaviors through specific and non-specific pathways (see Aktar, Nikolic, and Bögels [44] for a review of the literature), and/or that mothers of children with GAD feel more anxious when their child is worrying and expressing uncertainty. Experimental studies need to be carried out in order to understand the mechanisms behind the transmission of SAD and GAD between father, mother and child, as well as the direction of effects (parent to child, child to parent or both). Next to the possibility of a different transmission from mother and father to the child, the child’s gender could also play a role in the transmission of ADs. It has been found that rates of ADs between mothers and daughters were higher than between fathers and sons [14]. However, a more recent study did not find gender effects between sons and daughters [46]. Thus, in order to get a better understanding of the transmission of ADs, future studies should not only consider the specificity of ADs for mothers and fathers, but also whether this differs between daughters and sons.

As with all studies, the current study is not without limitations. First, although the clinical sample size was large, not all odds ratios could be calculated. This study provided important information (i.e. no SAD fathers had children without SAD), but this issue should be examined further, for example by including a larger sample of children with SAD. Second, due to the large amount of children meeting criteria for several ADs, the interpretation of the current findings concerning the specific disorders is somewhat limited. Thus, in order to improve internal validity, only children with a single AD should participate; however, this will limit the generalizability to the field of child and adolescent psychiatry where comorbidity is the rule rather than the exception [26]. Third, the design of this bottom-up study only examined children with ADs and not children with other types of psychopathology, which limits the findings of specificity only to this AD sample, and it would be interesting to compare these rates to parents of children with other disorders. Fourth, the control group included children that met criteria for an AD, which raises the question whether this is a true control group. We have decided to keep the children with ADs in the control group, in order to support the external validity of our study. Nevertheless, the control group was smaller than the group of children with ADs, and it could be that the control group is less representative due to the recruitment through newspaper advertisements. Finally, the study design does not allow conclusions about the mechanisms that are underneath the observed transmission of anxiety (i.e. genetic and/or environment), nor about the direction of transmission.

Despite its limitations, this study represents a comprehensive evaluation of prevalence of ADs and specificity in mothers, fathers, and siblings of children referred with ADs. The study was one of the first to systematically include fathers and siblings, which adds to the existing knowledge of ADs running in families. Another strong point is that this study used structured diagnostic interviews for all family members and combined child and parent report for the child diagnoses. We have found that children with ADs are over twice as likely to have mothers and/or fathers with lifetime and/or current ADs. We have also found evidence for specificity of SAD and specificity of mother–child GAD. It has been shown that children of parents with ADs do less well in treatment than children of parents without ADs [7], and, in specific, children of mothers with SAD tend to do poorly compared to children of mothers without ADs [10]. In addition, this study showed that fathers and siblings of children with SAD reported high rates of SAD themselves. Siblings of children with ADs usually are not included in treatment and research (compared to siblings of children with special needs [17]), but it may be important to screen them for ADs as well and to consider involving them in treatment. Finally, treatment of SAD in children has been mentioned as least effective compared to other ADs [42]. Possibly, treatment of childhood SAD is less effective because parents’ SAD functions as a maintaining factor and parents are for example not able to facilitate social exposures because they lack certain social skills. Thus, it should be examined whether parents will also change when their child is treated or vice versa, especially regarding SAD.

## Footnotes

1 Additionally, the same analyses were performed with the 41 children of the control group without ADs. When leaving the children with ADs out of the control group, results did not change for having at least one parent with lifetime AD(s): (OR 2.18, 95% CI 1.03–4.62, *p* = 0.022), having at least one parent with current AD(s) (OR 3.27, 95% CI 1.25–8.55, *p* = 0.008), mothers’ lifetime AD(s) (OR 1.40, 95% CI 0.64–3.07, *p* = 0.200), mothers’ current AD(s) (OR 2.84, 95% CI 1.00–8.05, *p* = 0.025), fathers’ current ADs (OR 3.17, 95% CI 0.39–25.91, *p* = 0.141), and siblings’ AD(s) (OR 0.74, 95% CI 0.27–1.99, *p* = 0.274). The only change observed was for fathers’ lifetime AD(s), which became a trend to significance (OR 2.37, 95% CI 0.76–7.41, *p* = 0.070). When leaving the children with ADs out of the control group, results did not change for mothers’ lifetime depressive disorder (OR 1.13, 95% CI 0.47–2.74, *p* = 0.393), fathers’ lifetime depressive disorder (OR 0.99, 95% CI 0.32–3.01, *p* = 0.491), mothers’ current depressive disorder (OR 4.17, 95% CI 0.51–34.03, *p* = 0.092), and fathers’ current depressive disorder (OR 0.20, 95% CI 0.03–1.59, *p* = 0.064).
